# Degradation of Tetracycline on SiO_2_-TiO_2_-C Aerogel Photocatalysts under Visible Light

**DOI:** 10.3390/ma15051963

**Published:** 2022-03-07

**Authors:** Jian Wei, Pinghua Zhu, Peixin Chen

**Affiliations:** 1 Audit Office, Changzhou University, Changzhou 213164, China; wj21089@163.com; 2 Department of Civil Engineering, Changzhou University, Changzhou 213164, China; 00009208@cczu.edu.cn

**Keywords:** hydrothermal synthesis, SiO_2_-TiO_2_-C aerogel, visible light photocatalysis

## Abstract

SiO_2_-TiO_2_-C aerogel photocatalysts with different carbon loadings were synthesized by using sol-gel chemistry. The anatase crystal and nonmetal carbon dopant were introduced during the sol preparation and formed by hydrothermal treatment, which can simultaneously enhance the adsorption ability and visible light photo-activity. A high surface area (759 g cm^−3^) SiO_2_-TiO_2_-C aerogel composite can remove up to 80% tetracycline hydrochloride within 180 min under visible light. The characterization of the gel structures shows that the homogeneous dispersion of O, Si, Ti and C in the skeleton, indicating that hydrothermal synthesis could provide a very feasible way for the preparation of composite materials. n(C):n(Ti) molar ratio of 3.5 gives the best catalytic performance of the hybrid aerogel, and the cyclic test still confirms over 60% degradation activity after seven use cycles. All catalysis reaction followed the pseudo-first-order rate reaction with high correlation coefficient. The electrons and holes in the compound could be effectively restrained with doping proper amount of C, and ESR results indicate that the oxidation process was dominated by the hydroxyl radical (•OH) and superoxide radical (•O_2_^−^) generated in the system.

## 1. Introduction

Tetracycline (TC) is widely used in the medical industry and ultimately discharged through human waste to municipal wastewater plants [1]. Conventional sewage treatment processes are difficult to remove completely due to its stable chemical properties [2], which poses a great risk to human health [3,4]. In the past ten years, the pollution of TC was gradually valued by environmental scientists. The research on its degradation and removal technology was listed as a key environmental remediation project by many countries [5,6,7,8,9].

Based on the characteristics of low cost, high chemical stability and optical stability, and no secondary pollution, the photocatalytic technology represented by TiO_2_ is considered as a desirable method to deal with environmental pollution and an important way to solve the global energy crisis [10,11]. However, as a single component oxide, TiO_2_ still has some problems in practical application. The band gap width of the TiO_2_ is wider (3.2 eV). Therefore, the ultraviolet light (4%), which occupies only a few parts of sunlight, can not be used to absorb the visible wavelength and can not respond to visible light. High recombination rate of photon-generated carrier and low quantum efficiency, which leads to the decrease in photocatalytic activity [12,13]. In the visible area, the photocatalytic activity of titanium dioxide attracted significant research interest because visible light accounts for about 45% of the solar energy. The results show that the doping modification of TiO_2_ crystals by metal [14], composite semiconductor [15] and nonmetal [16] can enlarge the light absorption range, inhibit the photo-electron-hole pair recombination, and improve the photocatalytic performance effectively.

The transition metal ions and the rare earth metal ions are the most widely studied metal ions at present [17,18]. The charge transitions between the d electrons of the transition metal ions and the conduction bands or valence bands of the TiO_2_ can improve the optical quantum efficiency and the photocatalytic effect ability. Rare earth metals have a unique 4f electronic structure, which can be transferred between f-f and f-d configuration to become the trapping traps of optical electrons or cavities, but also can cause lattice distortion from the formation of oxygen vacancies to reduce the forbidden band width and broaden the spectral absorption range [19]. The addition of narrow band gap semiconductors such as ZrO_2_ [20], SiO_2_, WO_3_ [21], or Al_2_O_3_ [22], is also considered as an effective way to improve the thermal stability and visible light photocatalytic activity of TiO_2_. Among them, SiO_2_–TiO_2_ materials have been widely studied in the field of photocatalysis because they have higher photocatalytic activity than pure TiO_2_. Si^4+^ ions mainly exist in the form of Ti–O–Si structure on the surface of TiO_2_ particles, forming surface state energy levels in the certain regions of the conduction band [23]. The electron was transferred from the valence band to the surface state level in illumination, which stimulated the visible absorption effect. The surface hydroxyl and redox properties of catalytic agent under visible light is enhanced by doping silica material [24]. The results show that when doping nonmetal, its valence band rises and produces low band gap energy, thus improving catalytic efficiency [25]. Since Sato presented nitrogen-doped titanium dioxide as a visible photocatalytic performance in 1986, the study of nonmetallic doping TiO_2_ did not attract enough attention. Until 2001, Rasahi et al. [26] confirmed that N atoms replaced O atoms to form a hybrid orbit in the lattice of N-TiO_2_ materials, which causes the red shift of the absorption spectrum of TiO_2_ composites. Subsequently, other nonmetals such as S, C and P have also been incorporated into a TiO_2_ matrix for visible light-activated material. In a report by a group, the residual S was shown to occupy O sites in TiO_2_ and the band gap lowering was attributed to the mixing of S 3p and O 2p states [27]. Another group has indicated that C-doped TiO_2_ photocatalyst with high specific surface could be prepared by furfural as carbon source and the main reason for the response of visible light was that the carrier could transfer on both TiO_2_ and C simultaneously [28]. In conclusion, TiO_2_ is a general catalyst for photocatalytic degradation of many nonbiodegradable organic pollutants [29], but the pure TiO_2_ is only functional at UV radiation range. Doping TiO_2_ with nonmetals such as N, S or C extends the absorption wavelengths from UV to visible region, which has been ascribed to the introduction of localized electronic states in the band gap but will not influence the degradation selectivity of the TiO_2_ [30]. 

In the present study, high surface area charge transfer support material SiO_2_ aerogel was selected to hybrid with anatase TiO_2_ with a design target to improve the interactions between the pollutant and the catalyst; in addition, a nonmetal dopant carbon was applied for the enhancement of the system’s visible light response. All three components were prepared ‘one-pot’ in one aerogel composite by using the wet sol-gel chemistry, followed by hydrothermal treatment, in which the crystallization of TiO_2_ and carbonization of D-fructose were carried out simultaneously. The final SiO_2_-TiO_2_-C aerogel composite displays a high photocatalytic activity, which could efficiently reduce/remove the medical pollutants, i.e., tetracycline in this study, from the aqueous solution (by 80%) in 180 min. The micro morphologies, crystal structure, pore size distribution and chemical structures are intensively studied to compare the aerogel properties loaded with various amounts of carbon dopants, which correlate to the final photocatalytic performance. Finally, the catalytic mechanism of the newly developed aerogel composite catalyst was examined by studying the charge transfer and free radicals formation in the porous structure.

## 2. Experimental

### 2.1. Chemicals

Titanium tetraisopropoxide (Ti{OCH(CH_3_)_2_}_4_, Shanghai Macklin Biochemica Co., Ltd., Shanghai, China, 95%), tetraethoxysilane (TEOS, Si(OC_2_H_5_)_4_, Shanghai LingFeng Chemical Reagent Co., Ltd., Shanghai, China, 28.0%) and D-Fructose (C_6_H_12_O_6_, Sinopharm Chemical Reagent Co., Ltd., Shanghai, China) were used as titanium, silicon and carbon sources, respectively. Trimethylchlorosilane (TMCS, C_3_H_9_ClSi, Sinopharmynthes Chemical Reagent Co., Ltd., Shanghai, China, 98.0%), n-Hexane (C_6_H_14_, Chinasun Specialty Products Co., Ltd., Shanghai, China, 95.0%) and iso-propyl alcohol (IPA, Shanghai LingFeng Chemical Reagent Co., Ltd., Shanghai, China, 99.7%) were used for one-step solvent exchange and surface modification processtion process. Sulfuric acid (H_2_SO_4_, Sinopharm Chemical Reagent Co., Ltd., Shanghai, China, 95.0%) was added as a complexing agent. Distilled water, made by our laboratory, which was used as the solvent. All the chemical reagents were used as received.

### 2.2. Catalyst Preparation

The starting compositions of sample gels prepared are listed in Table 1. The compostion of SiO_2_-TiO_2_-C aerogel photocatalysts can be preformed with two parts. In the first place, TEOS was added into deionized water, the whole mixture was slightly stirred (200 rpm) for 1 h at normal temperatures and pressures. Thereafter, iso-propyl alcohol, deionized water and sulfuric acid were added to the above-mixed liquor and stirred for 1.5 h at 40 °C. Subsequently, Ti{OCH(CH_3_)_2_}_4_ was slowly dropped followed by stirring at a normal temperature for 2 h. Then, the above solution was allowed to stand for 1 h to prepare the SiO_2_–TiO_2_ wet gel. Second, the wet gel and fructose solution were put together in the reactor at 80 °C for 4 h, then the temperature of the reaction was raised to 180 °C within 7 min and kept as steady as possible for 12 h. SiO_2_-TiO_2_-C wet gel was taken out after the reaction completed, aged at normal temperatures for 24 h, modified with TMCS/IPA/n-Hexane(volume ratio of TMCS/IPA/n-Hexane = 1:0.3:1) solution which changed once every 24 h until the water was completely replaced, the gel blocks could be observed floating on the modified solution. The SiO_2_-TiO_2_-C aerogel was obtained after heat treatment at 50, 80, 150 and 180 °C for 2 h, respectively, in the oven.

### 2.3. Characterization

Powder X-ray diffraction (XRD). The crystal structure of the TiO_2_ was recorded on a Bruker APEX II DUO diffractometer equipped with Cu Kα radiation (λ = 1.5418Å).

Evaluation of the Brunauer–Emmett–Teller (BET) specific surface area. Nitrogen adsorption/desorption measurements were carried out by a Micromeritics TriStar II 3020 V1.03 (Atlanta, GA, USA) analyzer at 77.350 K. The surface area and pore volume of the sample were calculated using Brunauer-Emmett-Teller and Barrett-Joyner-Halenda methods, respectively.

SEM characterization. SEM analysis of all materials was performed on a Hitachi FE-SEM SU8000 instrument (Tokyo, Japan) at an accelerating voltage of 5 kV and a working distance of 9.1 mm.

Fourier transform infrared spectroscopy (FTIR). FTIR spectroscopy was recorded on a Nicolet iS50 instrument (Thermo Fisher Nicolet, Waltham, MA, USA).

X-ray photoelectron spectroscopy (XPS). XPS analysis was measured on an ESCALAB 250 photoelectron spectroscopy (Thermo Fisher Scientific Inc., Waltham, MA, USA) at 3.0 × 10^−10^ bar with monochromatic Al Kradiation.

UV–vis spectra analysis. UV–vis diffuse reflectance spectroscopy was recorded on UV-3600 UV–vis spectrometer, operated in the diffuse reflectance mode, for the wavelength in the range of 200–700 nm.

Electron Spin Resonance. ESR (Bruker A300, Billerica, MA, USA) was used to detect the active species of sample prepared by photocatalytic material.

### 2.4. Photocatalytic Measurements

The photocatalytic degradation test was conducted in an XPA-2 photochemical reaction apparatus. Under the action of the circulating cooling water and the electric fan, the entire photocatalytic reaction was performed under visible light (λ ≥ 420 nm) at about 30 °C. Take 50 mL TC solution with a concentration of 10 mg L^−1^ into a test tube, then turn on the light source for photocatalytic degradation reaction, sample 2 mL every 30 min, and the entire photocatalytic reaction lasts for 180 min. The sample was centrifuged at 6000 rpm for 10 min and the supernatant was stored for testing. In addition, 7 cycles of TC degradation tests were performed to investigate its optical stability. The photodegradation efficiency was calculated using the following equation:

(1) $$D=\left(C_{0}-C\right)/C_{0}$$

where *C*_0_ (mg·L^−1^) and *C* (mg·L^−1^) are values of concentration of dye solution at initial and time ‘*t*’, respectively.

The SiO_2_-TiO_2_-C aerogel catalytic redox reactions with TC proceed via pseudo-first-order kinetics [31]. The apparent rate constant (*k*) was determined from pseudo-first-order rate equation which is:

(2) $$ln\left(C_{0}/C\right)=kt$$

where *C*_0_ (mg·L^−1^) and *C* (mg·L^−1^) are values of concentration of dye solution at initial and time ‘*t*’, *k* is the apparent rate constant.

## 3. Results and Discussion

### 3.1. XRD Analysis

**Figure 1:**
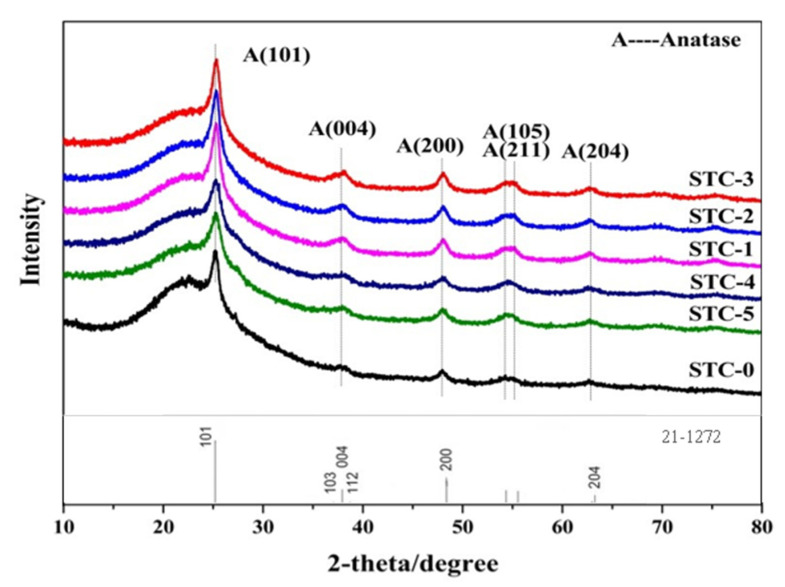
XRD diagrams of SiO_2_-TiO_2_-C aerogels with different C content.

Figure 1 displays the XRD patterns of the SiO_2_-TiO_2_-C aerogel photocatalytic materials with different C amounts. After heat treatment, six especial reflection peaks at 25.39° (101), 37.96° (004), 48.16° (200), 54.12° (105), 55.23° (211) and 62.56° (204) are ascribed to the anatase of TiO_2_ (JCPDS 21-1272), which proves the aerogels obtained possess anatase phase. Consequently, the incorporation of Si can effectively inhibit the transition of the TiO_2_ anatase to rutile phase [32]. The doped carbon element could move to the gap position or diffuse into the TiO_2_ lattice to form impurity [33,34]; the peak around 20° assigned to SiO_2_ is very wide due to the amorphous structure of SiO_2_ in the sample [23,24]. Compared with STC–0, the diffraction peaks of other photocatalysts are obviously enhanced, considering the relative intensities of the TiO_2_ and SiO_2_, indicating that C-doping increases the crystallinity of the samples. However, the position of the diffraction peak moves slightly toward the low angle, which may be caused by the substitution of the C atom for the O atom or Ti in the TiO_2_ lattice, thus increases the atom’s distance.

### 3.2. SEM and BET Analysis

**Figure 2:**
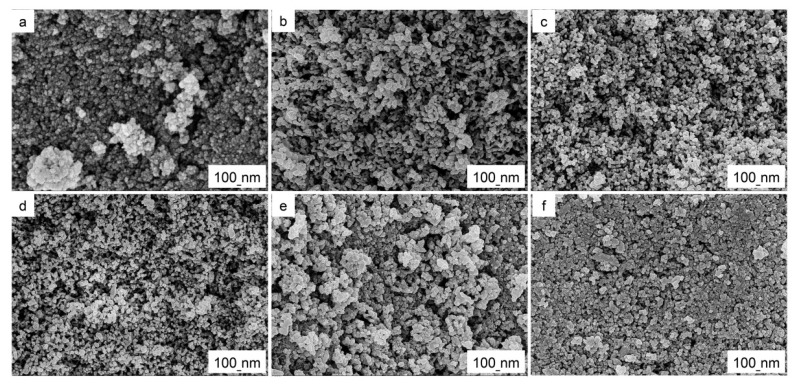
(**a**–**e**) SEM images: (**a**) STC–0, (**b**) STC–1, (**c**) STC–2, (**d**) STC–3, (**e**) STC–4 and (**f**) TC-5.

Figure 2a–f analyze the morphology of SiO_2_–TiO_2_ and five different SiO_2_-TiO_2_-C aerogel photocatalysts. All six samples are nanostructures. The morphology of the SiO_2_-TiO_2_-C aerogel is significantly more compact and smaller than the SiO_2_-TiO_2_ aerogel. This suggested that C-doping improve the photocatalytic activity of SiO_2_–TiO_2_ aerogels, and the catalytic performance of STC–3 may be the strongest [35], followed by TC degradation tests to further verify. With the increase of C content, the gel morphology changes from isolated macroporous structure (Figure 2b), through co-continuous structure (Figure 2c,d) to particle aggregates (Figure 2e,f). In other words, when the amount of C is few, aerogels with nanometer-sized pores are obtained; when the amount of C is large, serious agglomeration of internal structures occurs. The possible reason is that the driving force for agglomeration comes from the high surface free energy and large capillary force of the SiO_2_-TiO_2_-C aerogel. Aerogel has a large specific surface area and accordingly has a very high surface free energy. A large number of capillaries in the gel adsorb the dispersion medium in order to reduce its surface free energy [36]. During modification, colloidal particles coalesce due to the effects of surface energy and capillary forces, which resulted in hard agglomeration.

**Figure 3:**
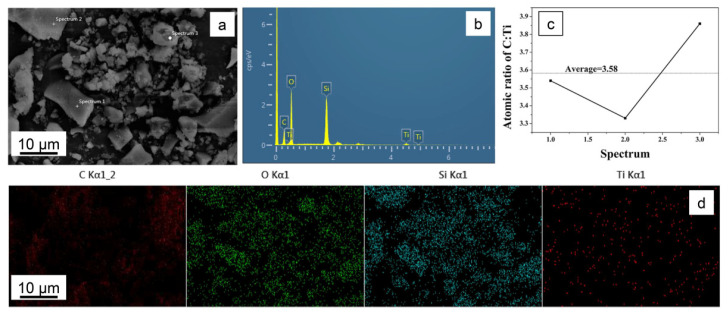
SEM image and (**a**–**d**) two-dimensional maps detected by EDX for O, Si, Ti, C, respectively, of sample STC–3.

Figure 3a–d show the EDX element mappings of STC–3. No aggregation is observed, which indicates that O, Si, Ti and C are dispersed uniformly in the skeleton. The C:Ti proportion is shown in Figure 3c, the average value is around 3.58:1, which is consistent with the stoichiometric design of 3.5:1. Compared with other synthetic techniques (sol-gel [37], impregnation [38], liquid-phase precipitation [39], etc.), hydrothermal synthesis can be more evenly doped, allowing the crystallites to grow according to their crystallization habit.

**Figure 4:**
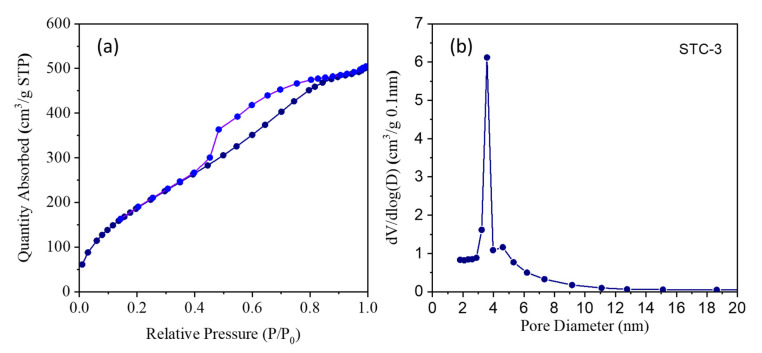
(**a**) The N_2_ adsorption–desorption isotherms, (**b**) the pore size distributions curves of STC–3.

Figure 4a shows N_2_ the adsorption–desorption isotherm of the STC–3 sample which according to the IUPAC classification corresponds type IV isotherms with H3-type hysteresis loop. There is a shifting process on the adsorption section with a relative pressure of less than 0.1, which indicates that there is a certain amount of microporous structure inside the sample. A sharp inflection in P/P_0_ > 0.48 related with capillary condensation is observed for the STC–3 sample which denotes a steep jump in the N_2_ adsorption volume indicating their mesoporous structures. The pore size distribution is mainly between 3–4.5 nm (Figure 4b, and the peak is sharp, indicating that the mesopores of the sample are relatively uniform. The results obtained at standard temperature and pressure (STP) indicate that the surface area is 759.28 m^2^/g, the mean pore diameter is 4.01 nm and the pore volume is 0.821 cm^3^/g.

### 3.3. FT-IR Spectra Analysis of Catalysts

**Figure 5:**
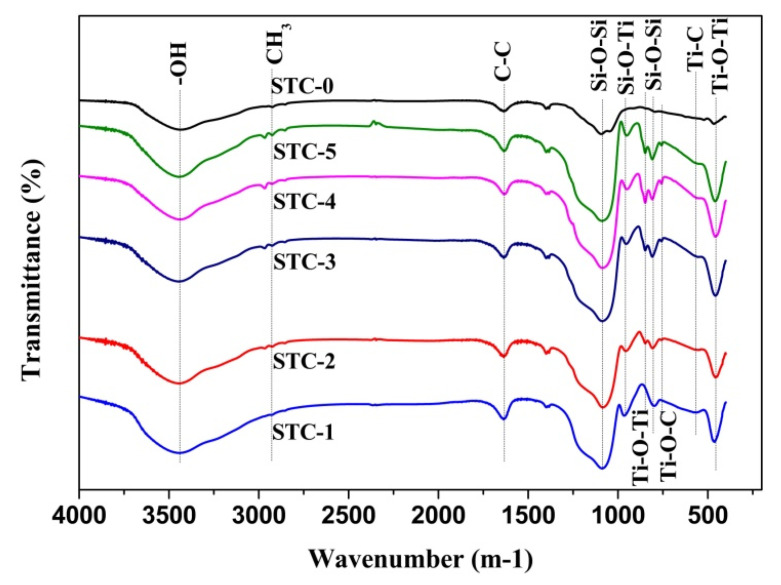
FT–IR spectra of aerogels: STC–0, STC–1, STC–2, STC–3, STC–4 and STC–5.

Figure 5 shows the FT-IR spectra of STC–0, STC–1, STC–2, STC–3, STC–4 and STC–5, respectively. The absorption bands at 1088, 808 and 459 cm^−1^ are observed in all spectra, which can be ascribed to the antisymmetric stretching vibration and symmetrical stretching vibration of Si–O–Si and stretching vibration of Ti–O–Ti, respectively [40,41,42,43]. A peak at 960 cm^−1^ corresponding to the stretching vibration of the Ti–O–Si bond is observed [44]. The vibrational feature at 3445 cm^−1^ is due to the surface OH groups [45]. The peaks of all TiO_2_—SiO_2_/C aerogels are relatively strong because C interacts with H, which caused the stretching and broadening of characteristic peaks of hydroxyl groups. The absorption peak near 2962 cm^−1^ corresponds to CH_3_ groups. The presence of the CH_3_ group has been attributed to the wet gel silanization effect [46] and shows hydrophobicity of the samples. The peak at 1621 cm^−1^, which is due to the vibration of C–C bonds, can confirm the successful doping of C. However, the sample STC–0 also has a weak peak at 1621 cm^−1^, which may be an impurity introduced during the preparation. Many significant peaks at 748 and 591 cm^−1^ are mainly related to the vibrations of Ti–O–C and Ti–C, respectively [47]. With the increase in C content, the absorption peak of Ti–O–C group is more and more obvious, and the intensity of the Ti–O–Ti bond absorption peak is accordingly enhanced.

### 3.4. XPS Analysis of Catalysts

**Figure 6:**
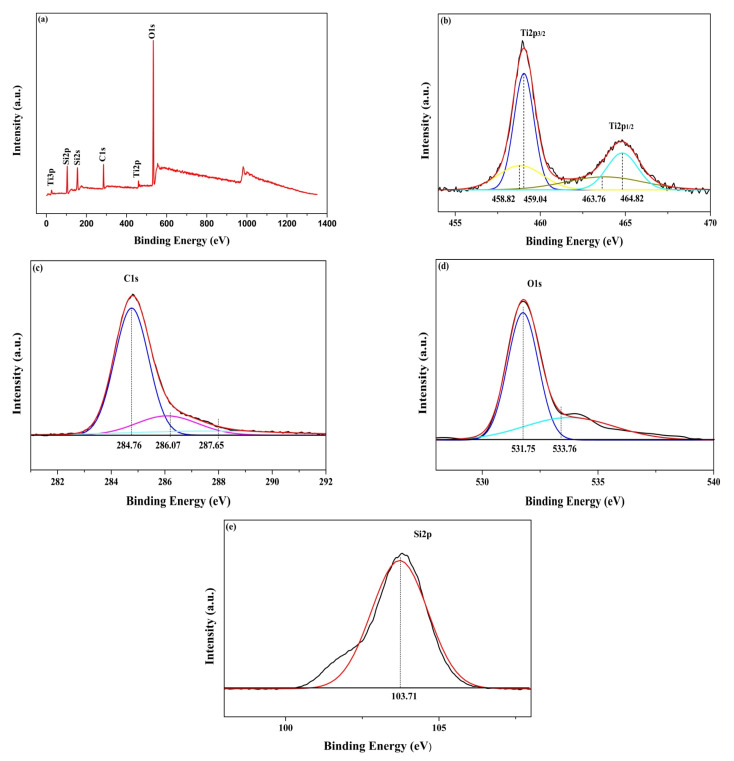
X-ray photoelectron spectroscopy of the SiO_2_–TiO_2_ aerogel STC–3, with survey spectra (**a**), high-resolution Ti 2p (**b**), C 1s (**c**), O 1s (**d**) and Si 2p (**e**).

XPS survey spectra of the as-prepared STC–3 is shown in Figure 6. Figure 6a is a schematic diagram of the full spectrum scan of the sample. The surface contains strong peaks such as Ti 2p, Si 2p, C 1s and O 1s, indicating that STC–3 contains four elements of Ti, Si, C, O, and the purity of SiO_2_-TiO_2_-C aerogel is higher. To further determine the valence state of each element in STC–3, high-resolution XPS studies were performed on Ti 2p, C 1s, O 1s and Si 2p (Figure 6b–e). The two peaks of Ti 2p can be resolved into two Gaussian peaks (Figure 6b). The binding energy peaks (464.82 and 459.04 eV) can be indexed to the 2p_1/2_ and 2p_3/2_ core levels of Ti^4+^, respectively, while the two peak energies at 463.76 and 458.82 eV are consistent with the characteristic Ti 2p_1/2_ and Ti 2p_3/2_ peaks of Ti^3+^ [48], respectively. Because the STC–3 sample is not doped by other elements, the possible defect states are responsible for the existence of oxygen vacancies and surface hydroxyl groups on TiO_2_ [49]; the peak of Ti 2p_3/2_ (459.04 eV) shows the Ti–O–C bond, which further proves that the carbon is integrated into the lattice and oxygen substitutes. The peaks at 286.07 and 287.65 eV (Figure 6c) are assigned to the C–OH and C=O bonds, indicating that the combination of C and O in TiO_2_ produces a large number of oxygen vacancies, which can be attributed to the carbon in the titanium dioxide crystal lattice [50]. The main peak of C 1s at 284.76 eV can be attributed to the elemental carbon on the surface of the sample, which may originate from environmental or dry residues. The O 1s signals of the STC–3 sample is shown in Figure 6d. The high-intensity peak situates at 531.75 eV, corresponding to Ti, Si and O atoms joined to form Ti–O–Si bond. The peak at the low intensity of 533.76 eV returns to C-O bond. Figure 6e shows a Si 2p XPS spectrum with peaks at 103.71 eV, indicating the formation of a Si–O–Si bond. These results show that the SiO_2_-TiO_2_-C aerogel photocatalyst was successfully prepared by hydrothermal method, and no other impurities appeared. The quantitative analysis results of XPS are shown in Table 2. SiO_2_-TiO_2_-C (3.5) photocatalyst consists of 5.3% Ti, 25.07% Si, 50.67% O and 18.96% C. The ratio of C:Ti is 3.577, which is basically consistent with the analysis of EDX result.

### 3.5. Photocatalytic Activity of Catalysts

#### 3.5.1. UV–Vis Diffuse Reflectance Spectroscopy and Energy Level Spectrum

**Figure 7:**
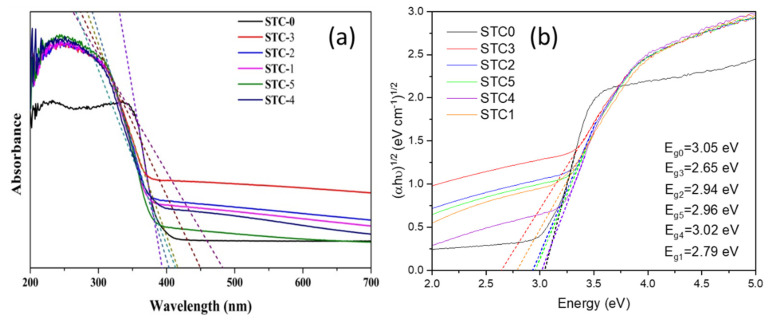
UV−vis absorption spectra (**a**) and the plots of (*αhν*)^1/2^ vs. the energy of absorbed light of (**b**) hybrid aerogels.

Figure 7 shows the UV-vis absorption spectra of the six samples, respectively. The band gap energy of the carbon-doped photocatalysts is significantly reduced, causing red shifting of the absorption edge to 400–500 nm, and extending the absorption wavelength response range to the visible light region. Carbon doping may lead to an energy-level disorder of the hybrid orbital between the C 2p and the O 2p [51], which may be caused by the partial substitution of C atom for O atom. This promotes the micro-transformation of the TiO_2_ lattice to form a new energy level with a smaller band gap. The new energy level can undergo electron transition under light irradiation of λ ≥ 387 nm (visible light). In addition, a small amount of carbonized material deposited on the surface of TiO_2_ will also have a sensitization effect on absorption of visible light, which can reduce the band gap energy from 3.05 eV to 2.65 eV. The band gap is confirmed from the Tauc’s Equation (3), and the (*αhυ*)^1/2^ was plotted against the photon energy (*hυ*), shown in Figure 7b. This indicates a red shift after carbon doping and a narrower band gap.

(*αhυ*)^1/*n*^ = *β*(*hυ* − *E_g_*) (3)

where *β* is a constant called the band tailing parameter, *E_g_* is the energy of the optical band gap and *n* is the power factor of the transition mode, which is dependent upon the nature of the material, here *n* = 2, whether it is crystalline or amorphous.

#### 3.5.2. Photocatalytic Degradation Performance Analysis

**Figure 8:**
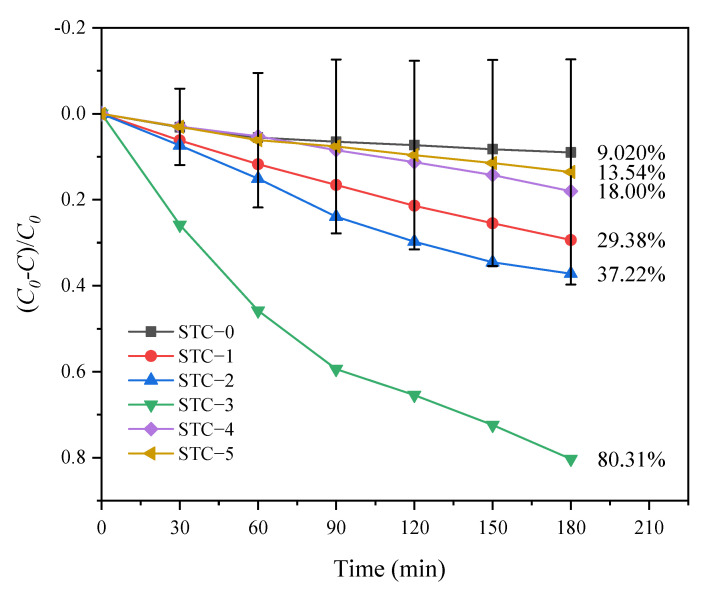
Catalytic Degradation of TC (10 mg/L) by Six Samples in Visible Light.

As shown in Figure 8, the photocatalytic activities of SiO_2_–TiO_2_ aerogel and five different SiO_2_-TiO_2_-C aerogels under visible light irradiation followed the order: STC–3 (80.01%) > STC–2 (37.15%) > STC–1 (29.37%) > STC–4 (18.02%) > STC–5 (13.5%) > STC–0 (9.09%) (Equation (1)). After doping with C, the degradation performance of the material can be significantly improved, mainly because the C-doping can reduce the band gap of TiO_2_ (Figure 7b), which increases the material’s ability to absorb visible light, enhances electron-hole generation and accelerates the redox reaction 28. In addition, C-doping has a great influence on the photocatalytic activity of TiO_2_. With the increase in the doping ratio, the degradation performance of TC becomes stronger and stronger; when n(C):n(Ti) is 3.5, the degradation of TC by STC–3 is optimal, reaching a maximum of 80.01% at 180 min, by using Equation (1). The further increase in doping of the C reduces the photocatalytic performance of the SiO_2_-TiO_2_-C aerogel, which may be caused by a hypothesis of the pore blockage. Additionally, the excessive doping of C could cause an increase in e^−^ and h^+^ recombination rate because the average distance between the trap sites decreases, which results in the decrease in catalytic efficiency [52].

#### 3.5.3. Kinetics Analysis

**Figure 9:**
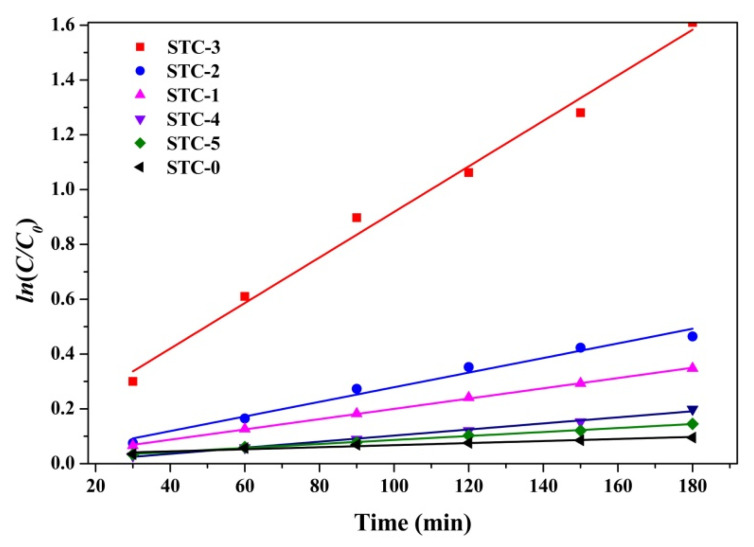
Six kinds of samples for the photocatalytic kinetic equation.

The simplified pseudo-first-order kinetic model of Langmuir-Hinshelwood (Equation (2)) is used to attempt a description of the apparent rate constant of the degradation process of TC. The results are shown in Figure 9 and Table 3. The apparent rate constants are 0.00038, 0.00187, 0.00267, 0.00831, 0.00111 and 0.00073 min^−1^, respectively. All fitting lines exhibited correlation coefficient (R-square) values more than 0.95. This indicated that the photodegradation process of TC within 180 min follows a good linear relationship. In addition, the rate constant k of STC–3 is about 21. Additionally, 4.4, 3.1, 7.5, and 11.4 times for other samples STC–0, STC–1, STC–2, STC–4 and STC–5, respectively. The results further confirmed that the prepared STC–3 has the best photocatalytic performance.

#### 3.5.4. Stability Analysis

**Figure 10:**
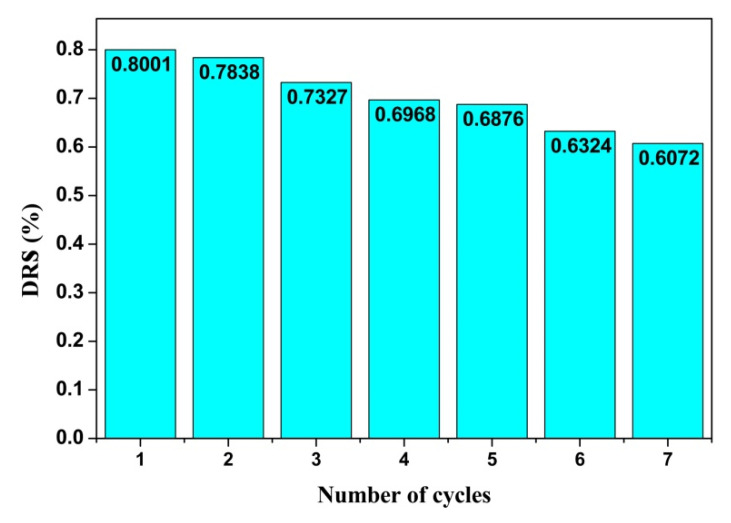
STC–3 TC degradation rate as a function of cycle number.

The stability analysis of the photocatalyst before and after the catalytic reaction is very important, and the stable catalyst can reduce the cost of the photocatalytic process to a great extent in the practical application process. The STC–3 with the strongest photocatalytic activity is selected as the object of investigation to perform the cyclic degradation TC test to evaluate the optical properties of SiO_2_-TiO_2_-C aerogels in order to verify whether the SiO_2_-TiO_2_-C aerogel prepared in this experiment is suitable for engineering promotion. The stability is shown in Figure 10. After seven cycles of experiments, the removal efficiency in the first cycle is 80.01%, and in the last cycle, it is 60.72%, a decrease of 19.29%, indicating that the photocatalytic material prepared in this experiment has good stability. The comparison between this paper and other work is shown in Table 4.

The comparison of different degradation results is shown in Table 4. It was clear that TiO_2_ composite aerogel has a significant degradation on some specific pollutants within 2–3 h, regardless of using UV light or Xe lamp as light source. However, the stability of removal efficiency was not considered in these studies. In this work, after seven cycles of removal experiments, the photocatalytic materials prepared in this experiment still has good stability.

### 3.6. Mechanism Analysis

#### 3.6.1. Analysis of Binding Mechanism

**Figure 11:**
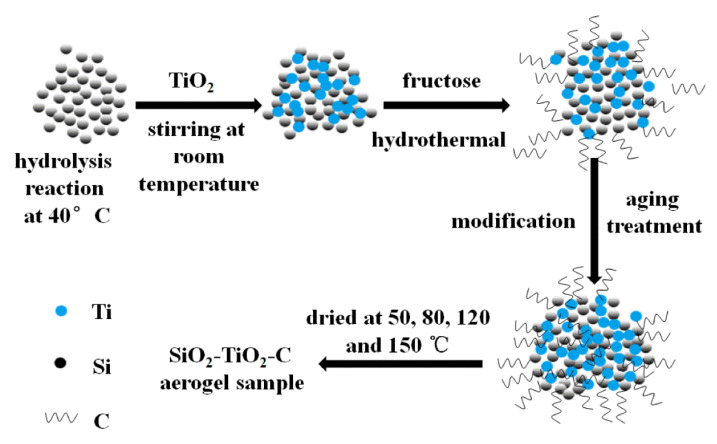
Scheme illustrating the synthesis of SiO_2_-TiO_2_-C composite aerogels.

Based on the characterization results of XPS, FT-IR, UV-Vis, BET, SEM and XRD, the formation mechanism of aerogel photocatalyst can be summarized as follows (Figure 11): on the one hand, the main form of Si atoms incorporated is the formation of Ti–O–Si bonds through chemical bonds and TiO_2_, which keeps the crystal structure stable; the introduction of SiO_2_ into the TiO_2_ crystal structure can effectively reduce the size of the crystal particles and increase the specific surface area of the composite photocatalyst. On the other hand, part of the incorporated C atoms enters into the TiO_2_ crystal lattice and replaces part of the O atoms to form Ti–C bonds. Some C atoms combine with TiO_2_ through chemical bonds to form Ti–O–C bonds, which enhances the structural stability of the photocatalyst. C atoms can introduce new hybridization energy levels into the TiO_2_ crystal lattice (orbital hybridization of C 2p and O 2p), which reduce the band gap energy of TiO_2_ and stimulate visible light absorption.

#### 3.6.2. Photocatalytic Mechanism Analysis

**Figure 12:**
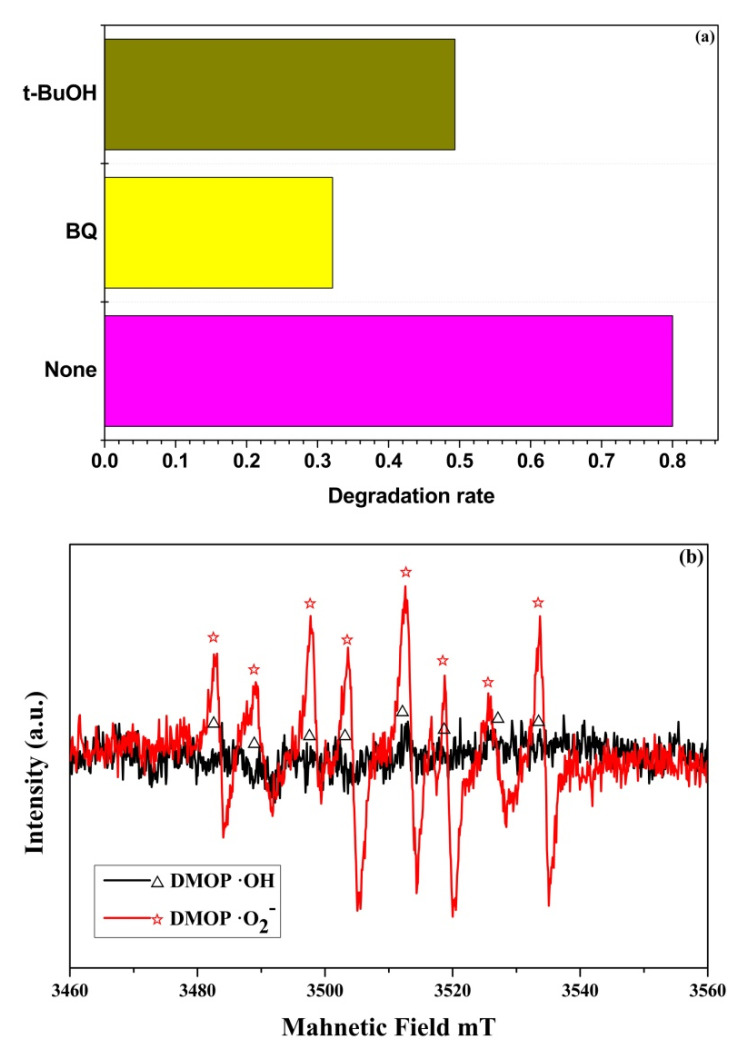
(**a**) Catalytic degradation of TC by STC–3 under different radical trapping conditions; (**b**) ESR spectra of STC–3.

**Figure 13:**
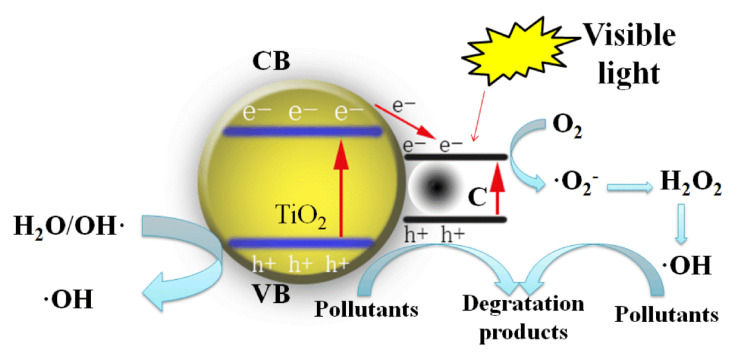
The proposed mechanism for the degradation of TC over SiO_2_-TiO_2_-C composite aerogels.

•O_2_^−^ and •OH were captured by benzoquinone (BQ) and tert-butyl alcohol (t-BuOH), respectively. The result is shown in Figure 12a. The degradation rate of TC is greatly reduced after the addition of BQ and t-BuOH in the system, which indicates that •O_2_^−^ and •OH play a key role in the photocatalytic degradation of TC. The STC–3 sample is tested for active species using ESR, as shown in Figure 12b. ESR is a modern separation technique used to determine shortlived free radicals. It can be useful to elucidate the mechanism of photocatalytic reactions 28. It can be seen that the characteristic peak intensity of •O_2_^−^ is significantly stronger than •OH after 60 s of illumination on STC–3 sample. It indicates that •O_2_^−^ is the main active substance produced by STC–3 under light. •OH also plays a role in improving photocatalytic efficiency, but it is not the main one. The enhanced performance of the photocatalyst was explained by the following mechanism (Figure 13). Under visible light irradiation, the carbon creates an additional band around the valence band of TiO_2_, which can be excited by visible light to generate carriers (Equation (4)). In the role of carbon atoms, electrons and holes are separated and migrated to different subsurface locations of particle, occurring redox reactions occur with •OH, HO_2_, and •O_2_^−^, etc. In detail, aerogel materials absorb energy equal or more than to the band gap, could also react directly with the O_2_ molecules, then form •O_2_^−^ active species (Equation (6)), positive holes remain in valence band (VB) (Equation (4)). The reaction between holes and H_2_O (Equation (5)), and the reaction between electrons and H_2_O_2_ (Equation (8)) both generate •OH. Finally, pollutant molecules are degraded by these active substances (Equation (9)).

In summary, the removal of the TC is mainly caused by two effects, (i) the absorption of the porous aerogel structure, and (ii) the photocatalytic degradation by the TiO_2_ active sites. We consider that the optimal removal of pollutant TC is the equilibrium between those two main effects, the TC is absorbed by the porous structure, when the carbon doping increases, the visible light response of the composite is improved, and we could identify a higher degradation effect. Meanwhile, carbon doping shows a negative effect to reduce the surface area of the composite. This means that the pollutant sorption to the photocatalytic sites is reduced. The direct reflection is the peak degradation on the sample STC–3.

(4) $${\mathrm{SiO}}_{2}-{\mathrm{TiO}}_{2}-C+h\nu \to h^{+}+e^{-}$$

(5) $$h^{+}+H_{2}O\to •\mathrm{OH}+H^{+}$$

(6) $$O_{2}+e^{-}\to O_{2}{•}^{-}$$

(7) $$O_{2}{•}^{-}+2H^{+}\to H_{2}O_{2}$$

(8) $$H_{2}O_{2}+e^{-}\to •\mathrm{OH}+{\mathrm{OH}}^{-}$$

(9) $$O_{2}{•}^{-}+H_{2}O_{2}+•\mathrm{OH}\ldots +\mathrm{TC}\to \mathrm{degradation}\;\mathrm{products}$$

## 4. Conclusions

In summary, SiO_2_-TiO_2_-C aerogel is synthesized by a hydrothermal synthesis method; C content has an important effect on improving the photocatalytic properties of SiO_2_-TiO_2_-C aerogel. When n(C):n(Ti) is 3.5, the sample has the best influence on the crystal structure and photocatalytic performance. Catalytic degradation studies for SiO_2_-TiO_2_-C aerogel demonstrate that the STC–3 catalysts can efficiently degrade 80.01% of TC dye in 180 min of illumination time and can retain high stability and reusability. The active species for photocatalytic degradation of TC are •O_2_^−^ and •OH. On the basis of this study, it can be anticipated that low-cost and high-performance SiO_2_-supporting composites for self-cleaning materials, such as self-cleaning ceramics, self-cleaning glass, self-cleaning cement-based materials, etc., may be fabricated through rationally designing the structure of composites.

## Figures and Tables

**Table 1:** Starting Compositions of the Samples.

Sample	TEOS (g)	Titanium (g)	H_2_O (g)	H_2_SO_4_ (g)	IPA (g)	C_6_H_12_O_6_ (g)	N(C):n(Ti)
STC–0	4.67	1.28	2.22	0.04	5.26	0	0
STC–1	4.67	1.28	2.22	0.04	5.26	0.40	1.5
STC–2	4.67	1.28	2.22	0.04	5.26	1.21	2.5
STC–3	4.67	1.28	2.22	0.04	5.26	2.02	3.5
STC–4	4.67	1.28	2.22	0.04	5.26	2.83	4.5
STC–5	4.67	1.28	2.22	0.04	5.26	3.65	5.5

**Table 2:** The atomic percentage of each element in STC–3 sample.

Sample	Ti	Si	O	C	C:Ti
STC–3	5.3%	25.07%	50.67%	18.96%	3.577

**Table 3:** First-order kinetic parameters of TC degradation by visible light in six samples.

	STC–0	STC–1	STC–2	STC–3	STC–4	STC–5
R-square	0.95058	0.99925	0.9771	0.98878	0.9923	0.99715
K (min^−1^)	0.00038	0.00187	0.00267	0.00831	0.00111	0.00073

**Table 4:** The comparison with other work.

Literature	Preparation	Aerogel	Target Pollutants	Light Source	Degradation
Zhao et al. [53]	Sol-gel	TiO_2_/SiO_2_/Ag terna-ry composite aerogel	oxytetracycline	UV light	36–66% in 120 min
Xu et al. [54]	Sol-gel	TiO_2_ polymethylsil-sesquioxane aerogel	Tetracycline hydrochloride	UV light	98% in 180 min
Shen et al. [55]	Sol-gel	Three-dimensional graphene aerogel	Tetracycline	Xe lamp	99.8% in 160 min
Y.M. Hunge et al. [56]	Water-based precipitation	TiO_2_@nanodiamond composites	Bisphenol A	UV light	100% in 100 min

## Data Availability

Not applicable.
